# A novel rejuvenation approach to induce endohormones and improve rhizogenesis in mature *Juglans* tree

**DOI:** 10.1186/s13007-018-0280-0

**Published:** 2018-02-09

**Authors:** Hao Liu, Ying Gao, Xiaobo Song, Qingguo Ma, Junpei Zhang, Dong Pei

**Affiliations:** 1 State Key Laboratory of Tree Genetics and Breeding, Research Institute of Forestry, Chinese Academy of Forestry, Beijing, 100091 China; 2 College of Horticulture and Landscape, Tianjin Agricultural University, Tianjin, 300384 China

**Keywords:** *Juglans*, Cutting, Rejuvenation, Endohormones, Rhizogenesis

## Abstract

**Background:**

*Juglans* is a difficult-to-root tree. In the present study, we successfully rejuvenated stock plants by grafting and then burying them horizontally.

**Results:**

Rooting rates of rejuvenated shoots were 98.1% 20 days after cutting. We recorded spatial and temporal variation in endogenous indole-3-acetic acid (IAA), abscisic acid (ABA), gibberellin A_3_ (GA_3_) and zeatin-riboside (ZR) under root induction. The four types of endohormones were mainly confined to the phloem sieve and companion cells (S&Cs) at the base of either rejuvenated or mature soft shoots. IAA and ABA levels were higher in rejuvenated shoots than in mature shoots, whereas the opposite was true for GA_3_ and ZR. During rooting induction, GA_3_ was the first hormone to be observed outside phloem S&Cs, followed by IAA, ABA and ZR. In rejuvenating soft shoots, IAA accumulated in the cross-sectional areas of the cambium and phloem, where root primordia were evident.

**Conclusions:**

The improvement in the rooting ability that was evident after rejuvenation most likely results a transformation of the plant to a juvenile form, from elevated IAA levels in phloem S&Cs and from a promotion of all four endohormones outside phloem S&Cs, in particular, from an accumulation of IAA in the cross-sectional areas of the cambium and phloem.

**Electronic supplementary material:**

The online version of this article (10.1186/s13007-018-0280-0) contains supplementary material, which is available to authorized users.

## Background

When trees age, their capacity to form adventitious roots becomes weaker and propagation via cuttings becomes increasingly difficult. From growers’ point of view, it is important to improve the adventitious root formation (ARF) capacities of mature trees. Some studies have shown that rejuvenation of mature trees or the induction of plants to regress from maturity to the juvenile state may significantly improve ARF. There are various ways to do this [2], including repeated in vitro subculturing, heavy pruning, mound-layering, serial grafting, and etiolation [50, 53]. The rooting competences of *Sequoia sempivirens* and chestnut (*Castanea sativa*) micro-cuttings can be restored by serial micro-grafting onto juvenile rootstock in vitro [19, 23]. The rooting abilities of teak (*Tectona grandis*) and common walnut (*Juglans regia* L.) can be improved by stock plant etiolation and exposure to periods of darkness in vitro [25, 38], respectively. In general, walnuts (genus *Juglans*) are recognized as being difficult-to-root species and even more difficult to propagate from cuttings.

Juvenile and mature trees differ in terms of phenotype, organizational structure and physiology [8, 42]. Rejuvenation restores the juvenile features of trees, for example, by increasing the activities of esterases and peroxidases [21], and by improving photosynthetic and respiratory rates [22]. Endohormones play important roles in tree rejuvenation. For example, cytokinins and gibberellins can induce rejuvenation and maintain trees in a juvenile state [16, 39]. Huang et al. [24] found that rooting abilities of successive generations of *Buxussinica* var. *parvifoli* cuttings were associated with changes in the levels of endohormones, including indole-3-acetic acid (IAA), abscisic acid (ABA) and gibberellin A_4_ (GA_4_). Negishi et al. [34] suggested that IAA/ABA levels reflect the extent of the juvenile phenotype in vitro and the rooting abilities of tender stems. Chang et al. [5] found that glycine-rich RNA-binding proteins accumulate at various rejuvenation stages of *Sequoia sempervirens.* Their expression is associated with the recovery of rooting ability and is regulated by both auxin and ABA [27, 28]. However, currently, the knowledge about (how or whether) the ARF of trees is improved by rejuvenation or the cytological action modes of various endohormones is quite limited.

The immune colloidal gold technique (ICGT) is a novel method for detecting endohormones in situ. In that method, colloidal gold serves as a marker of antigen–antibody interactions [10, 12–14, 18]. Ondzighi-Assoume et al. [36] used ICGT to show that ABA levels in the roots of *Arabidopsis thaliana* change in response to a nitrate treatment. The technique allows synchronous in situ monitoring of serial histological sections, thus being a useful immunohistochemical assay.

In the present study, we used rejuvenation to improve the rooting ability of soft shoots in walnut cultivars. We also subjected serial histological sections to various immunohistochemical assays to investigate spatial and temporal changes in the levels of endogenous IAA, ABA, gibberellin A_3_ (GA_3_) and zeatin-riboside (ZR). Moreover, we investigated the roles these hormones play to improve rooting by means of rejuvenation.

## Results

### Rooting of mature and rejuvenated soft shoots

After extensive preliminary studies, we found a method to make mature woody plants to be rejuvenated and rejuvenated soft shoots could induce rooting. The protocol is: the cultivar ‘Zhongningsheng’ [ZNS (*Juglans hindsii* × *Juglans regia*)] stock tree’s scions → grafted in 1-year-old plants → placed these grafted seedlings horizontally and culturing in greenhouse → harvest rejuvenated soft shoots → adventitious roots induction → rooting cultivation in greenhouse (detailed data reference method).

**Fig. 1:**
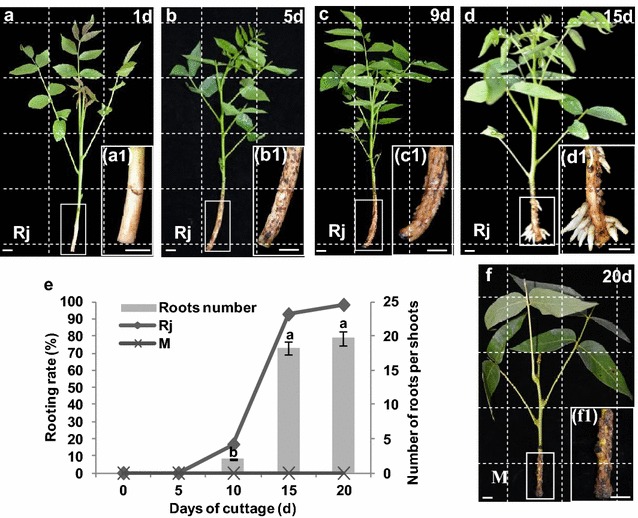
Rooting data and histological details of ARF in the basal region of soft shoots in walnut. **a** Rejuvenated soft shoots 1 day after cutting; *a1* color of epidermis was yellow-white. **b** Rejuvenated soft shoots 5 days after cutting; *b1* part of the soft shoots basal epidermis has browned. **c** Rejuvenated soft shoots 9 days after cutting; *c1* the adventitious roots tip have broken through out the periderm. **d** Rejuvenated soft shoots 15 days after cutting; *d1* the roots have broken through the epidermis and have assembled along the shoot. **e** Rejuvenated (Rj) soft shoot and mature (M) soft shoot rooting data, P < 0.05. **f** Mature soft shoots 20 days after cutting; *f1* no ARF but some callus was evident in the basal regions. Scale bars: 10 mm (**a**–**d**, **f**) (*a1*, *b1*, *c1*, *d1* was a twice magnified image respective of the white box in **a**, **b**, **c** and **d**)

By this method, under the same condition of rooting induction, rooting significantly differed between mature and rejuvenated soft shoots (Fig. 1). In rejuvenated soft shoots, adventitious roots broke through the epidermis after 10 days of induction (Fig. 1c, c1). By day 15, the rooting rate was 92.6% and the average root number per shoot was 18.3. Adventitious rooting occurred from the basal regions of rejuvenated soft shoots and was longitudinally distributed along the stem (Fig. 1d, d1). By day 20, the rooting rate had reached 98.1% and the average root number per shoot was 19.7 (Fig. 1e). In contrast, mature soft shoots exhibited no rooting up to day 20 after rooting induction. Only some callus was evident in the basal regions of parts mature soft shoots (Fig. 1f, f1).

### Histological observations

**Fig. 2:**
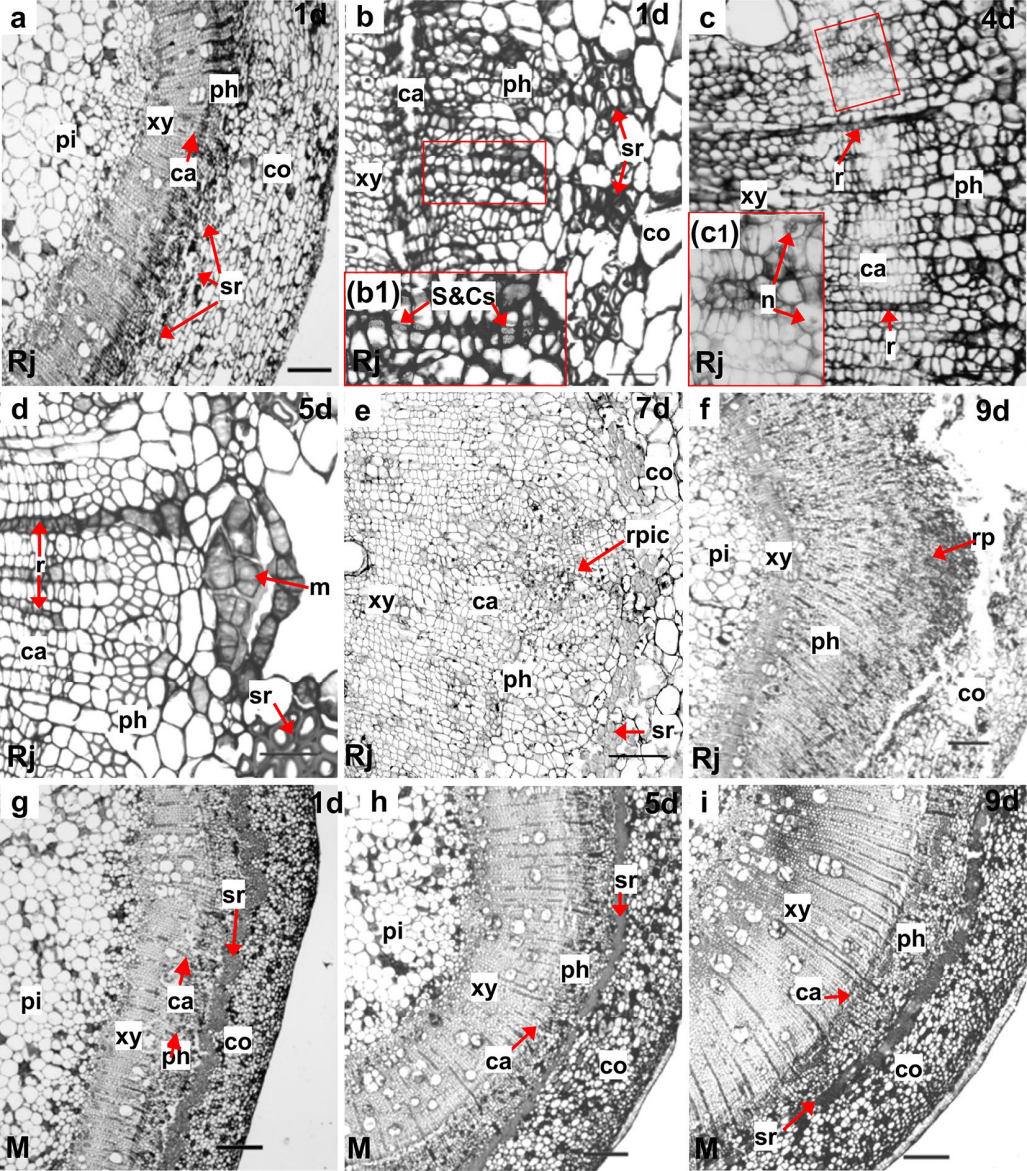
Histological details of ARF in the basal region of soft shoots in walnut. **a**–**f** Transverse sections prepared 1 day (**a**, **b**), 4 days (**c**), 5 days (**d**), 7 days (**e**), and 9 days (**f**) after rejuvenated soft shoots were placed in the rooting medium. **g**–**i** Transverse sections of mature soft shoots in walnut prepared 1 day (**g**), 5 days (**h**), and 9 days (**i**) after rooting induction. **a**, **b** The basal region of the rejuvenated soft shoot contains a discontinuous sclerenchymal ring (arrows). *b1* Showing sieve and companion cells (arrows). **c** Part of cambium is thickened and the nuclei are increased in size (*c1*: up arrow), some nuclei are in normal size (*c1*: right arrow). **d** Meristem cells located opposite ray cells are dividing. **e** The initial cells of the root primordia formed in the outermost region of the phloem. **f** Root primordia (the dome-like structures) grown into the cortex. **g** The basal region of a mature soft shoot is surrounded by a continuous sclerenchymal ring (arrows). **h** The xylem is thickened to about twice of the initial value. **i** The xylem becomes thicker over time; the other tissues do not exhibit significant changes. *ca* cambium, *co* cortex, *m* meristems, *n* nuclei, *ph* phloem, *pi* pith, *r* ray cell, *rp* root primordia, *rpic* root primordia initial cells, *sr* sclerenchyma, *S*&*Cs* sieve and companion cells, *xy* xylem. Scale bars: 200 μm (**a**, **f**–**i**); 100 μm (**e**); 50 μm (**b**–**d**) (*b1*, *c1* was a twice magnified image respective of the red box in **b**, **c**)

Figure 2 shows the histological differences between rejuvenated and mature soft shoots. Initially, the cortical cells in the basal regions of rejuvenated soft shoots were large and irregular, and separated by a discontinuous sclerenchymal ring from small parenchymal cells (Fig. 2a, b) located between the phloem and the cortex. However, in mature soft shoots, the phloem was surrounded by a continuous sclerenchymal ring, and the cortical cells were small and regular in shape (Fig. 2g). The anatomical structure of mature soft shoots was barely affected by cutting, although the xylem became thicker (Fig. 2h, i).

The histological structure of rejuvenated soft shoots was significantly different after IBA induction, as root primordia developed. On day 4, some cambium cells began to divide, and the cytoplasmic and nuclear densities became elevated (Fig. 2c). After 5 days of induction, dividing meristem cells were evident in the region of the phloem that faced the xylem rays (Fig. 2d). Two days later, initial root primordial cells formed from meristem cells (Fig. 2e). At the same time, small projections became visible on the peripheries of soft shoot bases. Subsequently, root primordia became evident in the phloem (Fig. 2f). On day 9, proliferations of the secondary phloem broke through the cortex, and the root primordia eventually became visible. Finally, the adventitious root primordia underwent complete development. A dome-shaped root cap formed and the vascular tissue became differentiated (Fig. 2f).

### Immunohistochemical localization of endohormones

**Fig. 3:**
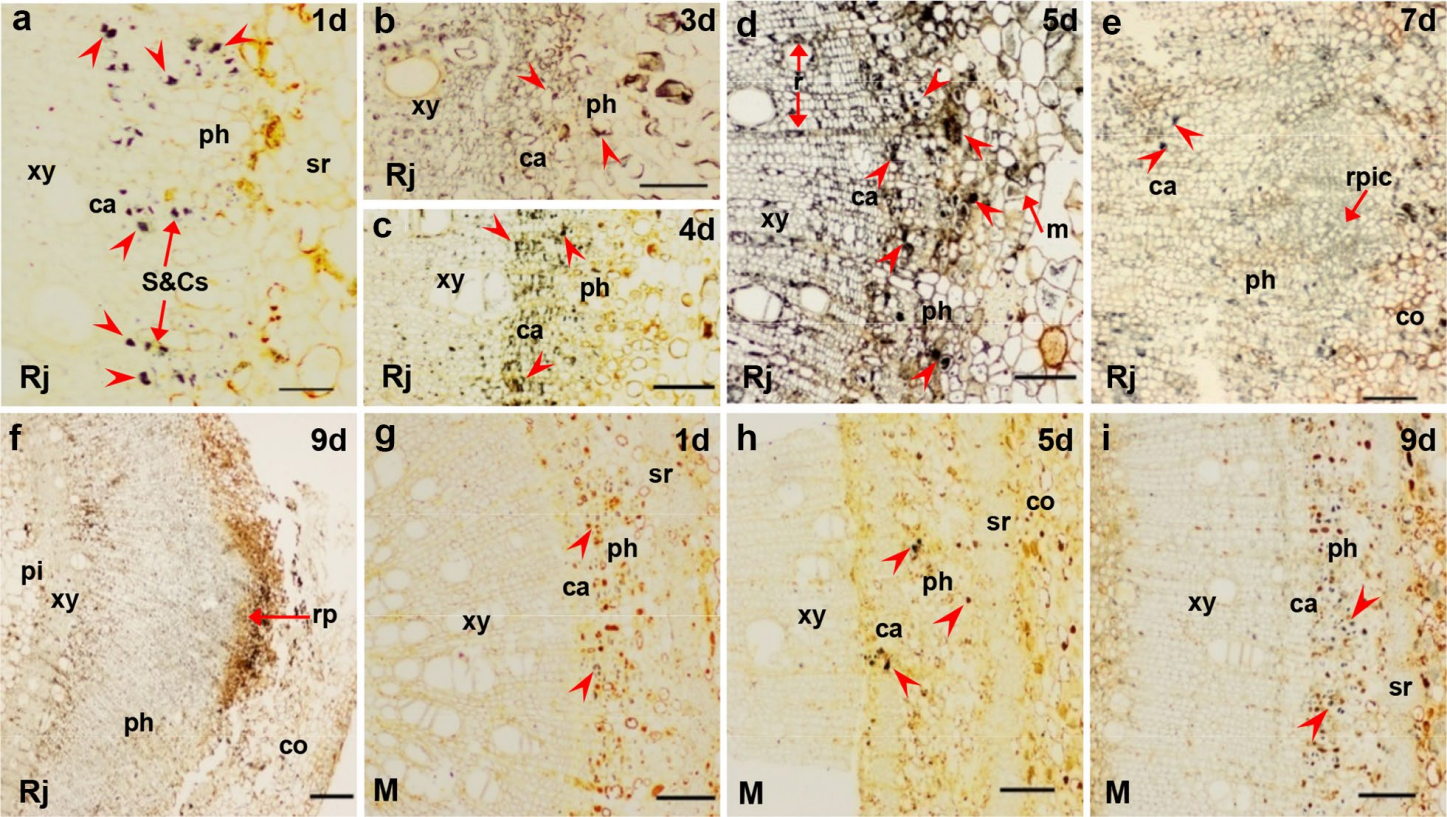
IAA immunolocalization in the stem bases of rejuvenated and mature soft shoots. **a**–**f** Transverse sections of rejuvenated soft shoots in walnut. **a**, **b** IAA is located in the stem 1–3 days after induction, but only in sieve cells and companion cells. **c** By 4 days, as the cambium thickens, IAA concentrates in this tissue. **d** By day 5, some meristem cells have divided and IAA is distributed in the meristems and ray cells. **e** IAA is present less in the initial cells of root primordia, part of cambium and S&Cs by 7 days. **f** The root primordia form by 9 days. IAA is distributed in the primordia, particularly in the tip regions. **g**–**i** IAA in the basal regions of mature soft shoots after 1 day (**g**), 5 days (**h**), and 9 days (**i**) of rooting induction. IAA signal is low at those times. The arrows indicate IAA signals. Arrowheads indicate the tissue structure. *ca* cambium, *co* cortex, *m* meristem, *ph* phloem, *r* ray cell, *rp* root primordia, *rpic* root primordia initial cells, *S*&*Cs* sieve and companion cells, *sr* sclerenchyma, *xy* xylem. Scale bars: 200 μm (**e**); 100 μm (**b**–**d**, **f**–**i**); 50 μm (**a**)

**Fig. 4:**
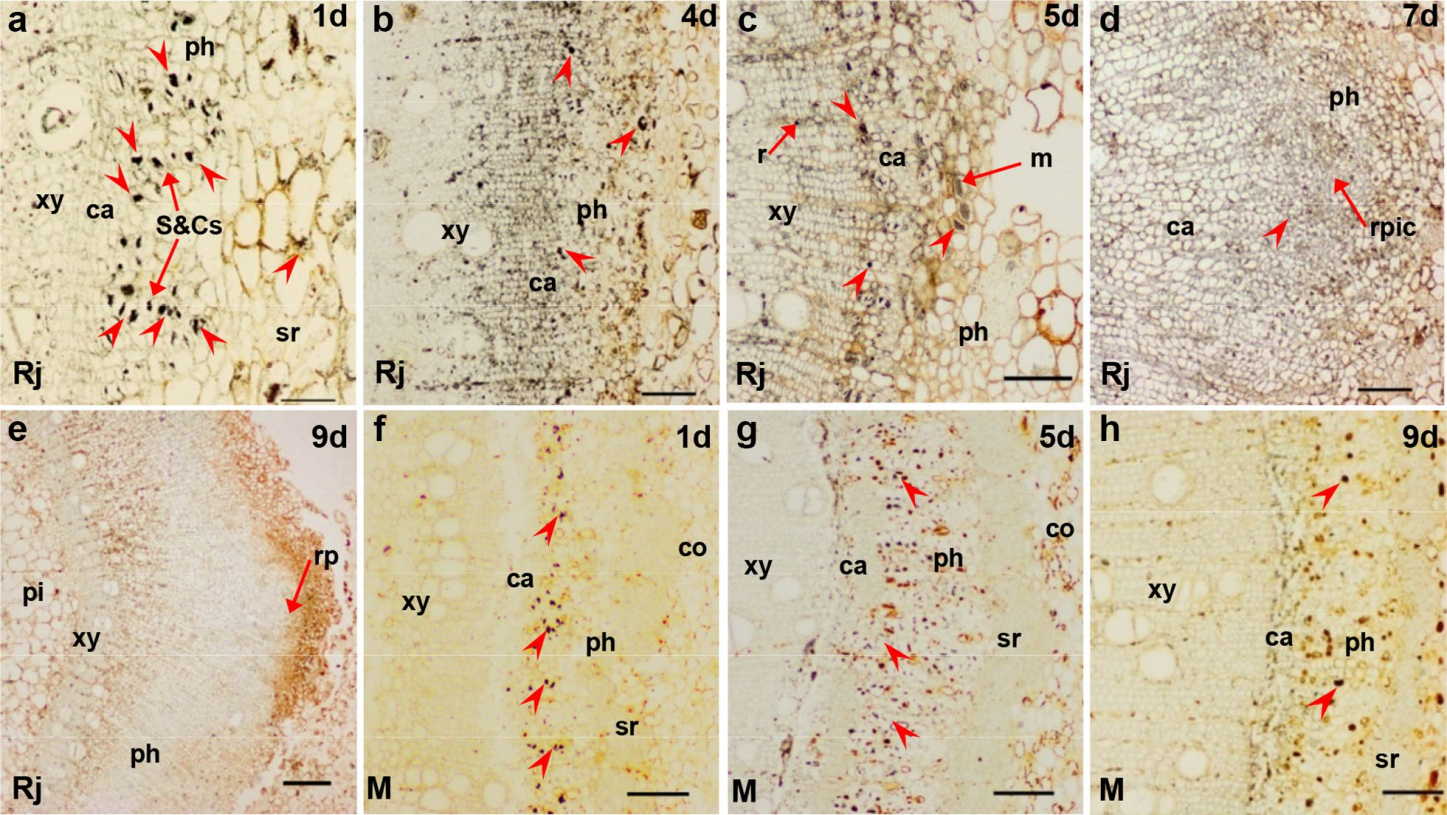
ABA immunolocalization in the stem bases of rejuvenated and mature soft shoots. **a**–**e** Transverse sections of rejuvenated soft shoots in walnut. **a** After 1 day of induction, ABA was found in S&Cs. **b** By 4 days, as the cambium thickened, ABA signal was concentrated in the cambium. **c** By 5 days, some meristem cells had divided and the ABA signal was located mainly in the meristem cells, part of cambium and ray cell. **d** By 7 days, some ABA signals were evident mainly in S&Cs and surrounding tissue of the initial cells of the root primordia. **e** The ABA level in root primordia formed by 9 days was minimal, comparison with supplementary (Additional file 1: Fig. s1 c), the dark color at the tip of the primordium were not ABA signals. **f**–**h** ABA signals from the bases of mature soft shoots; 1 day (**f**), 5 days (**g**), and 9 days (**h**). The temporal and spatial patterns of ABA expression did not change. The arrows indicate ABA signals. Arrowheads indicate the tissue structure. *ca* cambium, *co* cortex, *m* meristem, *ph* phloem, *rp* root primordia, *rpic* root primordia initial cells, *S*&*Cs* sieve and companion cells, *sr* sclerenchyma, *xy* xylem. Scale bars: 200 μm (**e**); 100 μm (**b**–**d**, **f**–**h**); 50 μm (**a**)

**Fig. 5:**
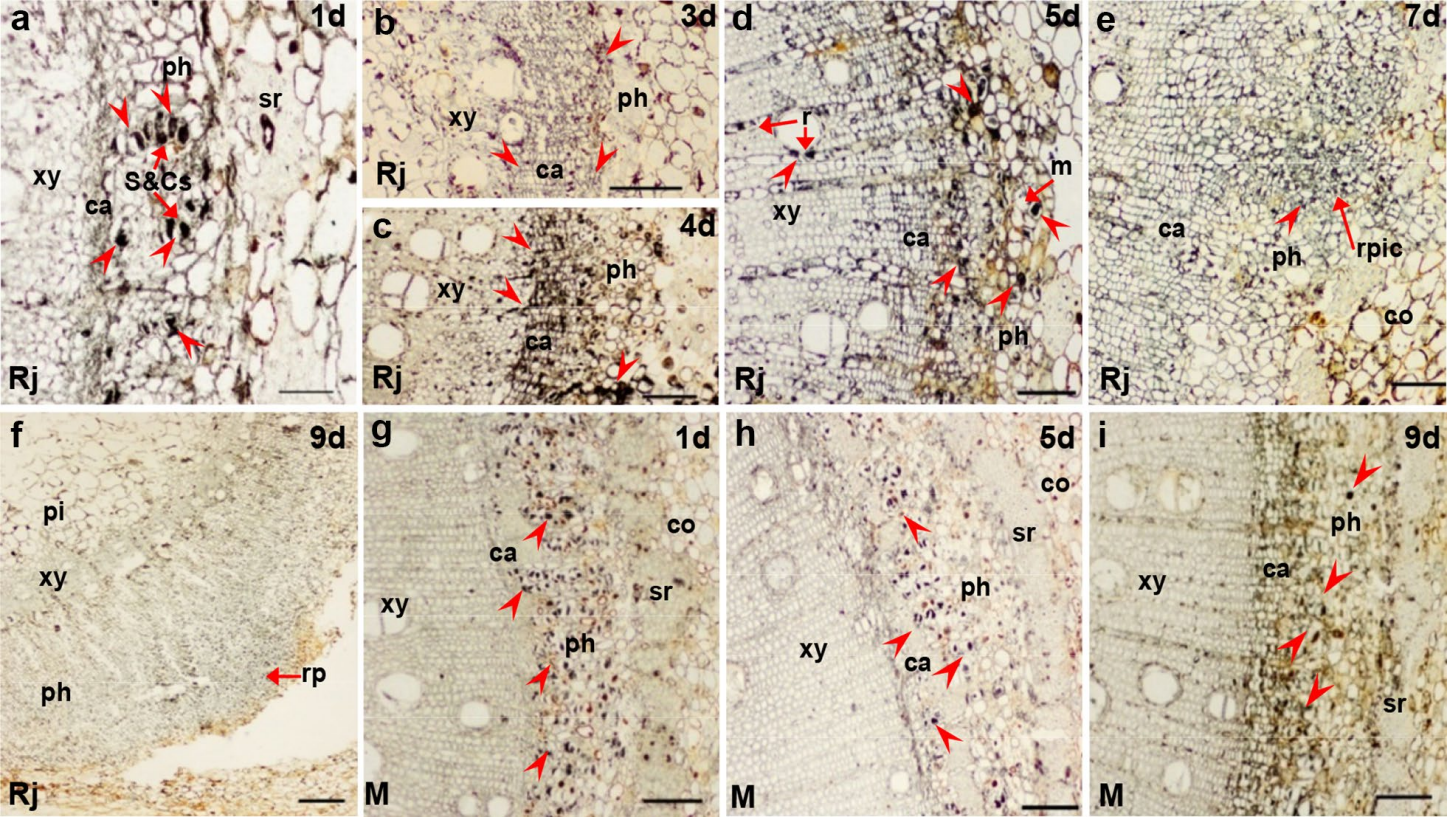
GA_3_ immunolocalization in the stem bases of rejuvenated and mature soft shoots. **a**–**f** Transverse sections of rejuvenated soft shoots in walnut. **a** GA_3_was located in the sieve and companion cells of the stem after 1 day of induction. **b** By 3 days, GA_3_ signal began to appear in the cambium, but remained mainly distributed in sieve and companion cells (S&Cs). **c** By 4 days, as the cambium thickened, the GA_3_ signal became concentrated in the phloem and cambium, particularly along the ray cells. **d** By 5 days, some meristems cells had divided and the GA_3_ signals were distributed among meristems, S&Cs and ray cells. **e** By 7 days, GA_3_ signals were evident in the initial cell root primordia, S&Cs and the cambium. **f** GA_3_ signals were widely distributed in the root primordia by 9 days. **g**–**i** GA_3_ signals in the bases of mature soft shoots on day 1 (**g**), 5 (**h**), and 9 (**i**),GA_3_ was located mainly in the S&Cs of the phloem, by 9 days (**i**), signal were evident in part of cambium. The arrows indicate the GA_3_ signals. Arrowheads indicate the tissue structure. *ca* cambium, *co* cortex, *m* meristem, *ph* phloem, *pi* pith, *r*ray cell, *rp* root primordia, *rpic* root primordia initial cells, *S*&*Cs* sieve and companion cells, *sr* sclerenchyma, *xy* xylem. Scale bars: 200 μm (**e**); 100 μm (**b**–**d**, **f**–**i**); and 50 μm (**a**)

**Fig. 6:**
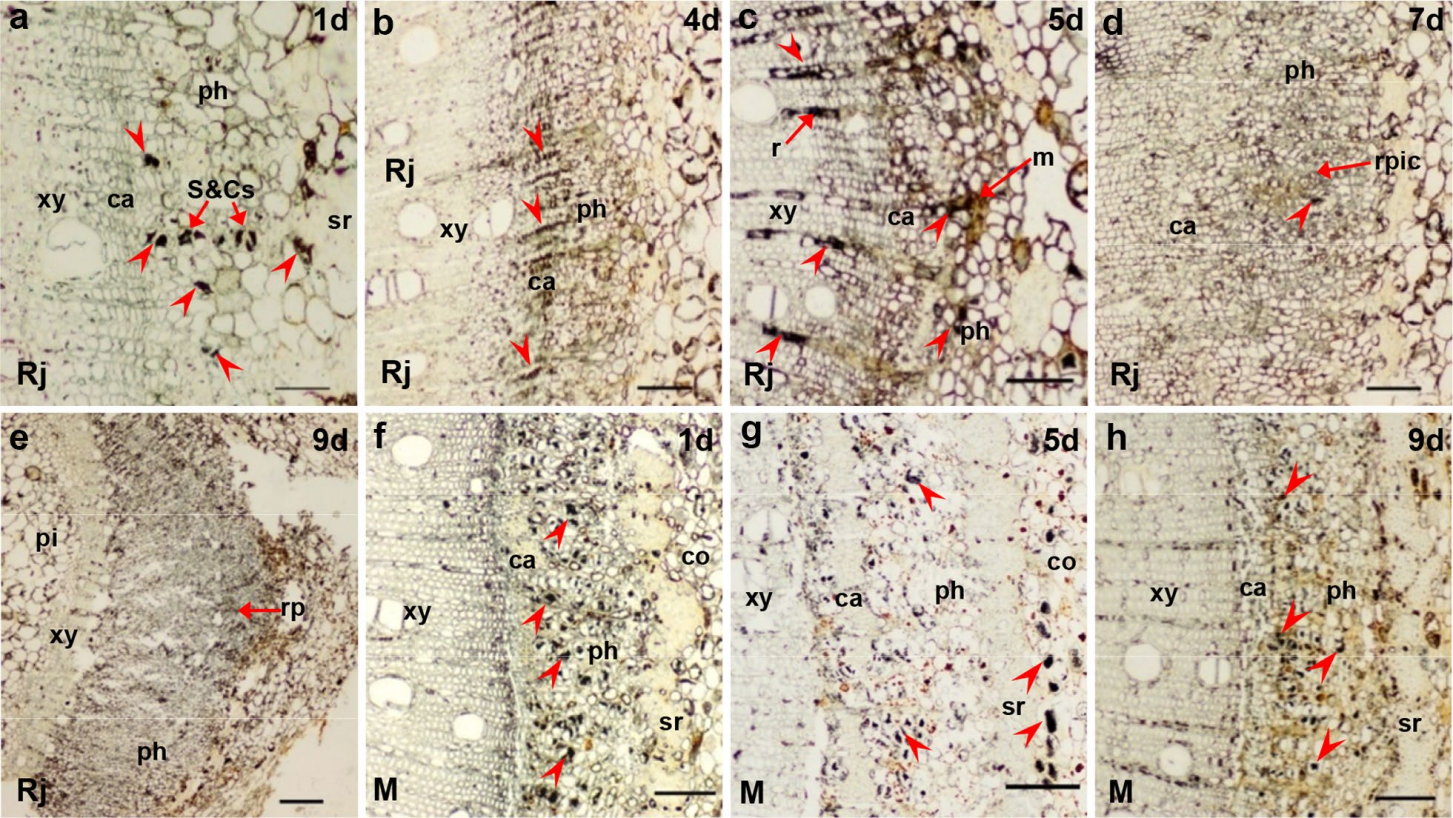
ZR immunolocalization in the stem bases of rejuvenated and mature soft shoots. **a**–**e** Transverse sections of rejuvenated soft shoots in walnut. **a** On 1 day of induction, ZR signals were located mainly in S&Cs. **b** By 4 days, as the cambium thickened, ZR became mainly concentrated in the phloem and cambium, particularly along ray cells. **c** By 5 days, some meristem cells had divided and the ZR signals were distributed among meristems, S&Cs and ray cells. **d** By 7 days, ZR signals were more evident in the initial cells of the root primordia, but less evident in cambium and S&Cs. **e** ZR signals were widely distributed in the phloem and root primordia by 9 days. **f**–**h** ZR was present more evident in S&Cs, but not so much in cambium of mature soft shoots on day 1 (**f**) and 5 (**g**). **f** ZR signals were observed between the xylem and the cambium by 5 days. **g** ZR signals were observed in the cortex by 5 days. **h** ZR signals were observed in ray cells by 9 days. The arrows indicate the ZR signals. Arrowheads indicate the tissue structure. *ca* cambium, *co* cortex, *m* meristem, *ph* phloem, *pi* pith, *r* ray cell, *rp* root primordia, *rpic* root primordia initial cells, *S*&*Cs* sieve and companion cells, *sr* sclerenchyma, *xy* xylem. Scale bars: 200 μm (**e**); 100 μm (**b**–**d**, **f**–**h**); and 50 μm (**a**)

Figures 3, 4, 5 and 6 show the immunological locations of IAA, ABA, GA_3_ and ZR in rejuvenated and mature soft shoots, and the temporal and spatial changes during adventitious root induction. In rejuvenated and mature cuttings, the four endohormones (IAA, ABA, GA_3_ and ZR) were consistently distributed mainly in the phloem sieve and companion cells (S&Cs) (Figs. 3a, g, 4a, f, 5a, g, 6a, f). During rooting induction, the endohormones of mature cuttings were always distributed mainly in phloem S&Cs, while other tissues contained small amounts of IAA, ABA and GA_3_, and even less ZR (Figs. 3h, i, 4g, h, 5h, i, 6g, h). However, the endohormone distributions in rejuvenated cuttings changed significantly during rooting induction, unlike what was noted in mature cuttings. At days 1–2 of rooting induction, all four endohormones were mainly distributed in phloem S&Cs. By day 3, GA_3_ was found in the cambium close to S&Cs of phloem (Fig. 5b). By day 4, the cambium had significantly thickened and contained all four endohormones, as did the adjacent ray cells (Figs. 3c, 4b, 5c, 6b). IAA accumulation was particularly obvious. IAA, ABA and GA_3_ were simultaneously present in some xylem ray cells located near the cambium. By day 5, adventitious root meristems had formed and the four endohormones were mainly distributed in the meristems, but also in the S&Cs. IAA, GA_3_, and ZR were also present in xylem ray cells (Figs. 3d, 4c, 5d, 6c). By days 6–7, initial root primordial cells had formed and all endohormones were present in these cells. ABA, GA_3_ and ZR were present also in the cambium and phloem (Figs. 3e, 4d, 5e, 6d). By days 8–9, adventitious root primordia of rejuvenated soft shoots had formed and some of them had broken through the epidermis. IAA and GA_3_ had accumulated close to the root cap. The cambium contained only a little ABA, as did also the root primordia and phloem. ZR became concentrated in the root primordia, and was present also in the phloem (Figs. 3f, 4e, 5f, 6e).

### Semi-quantitative analyses on endohormone levels

**Fig. 7:**
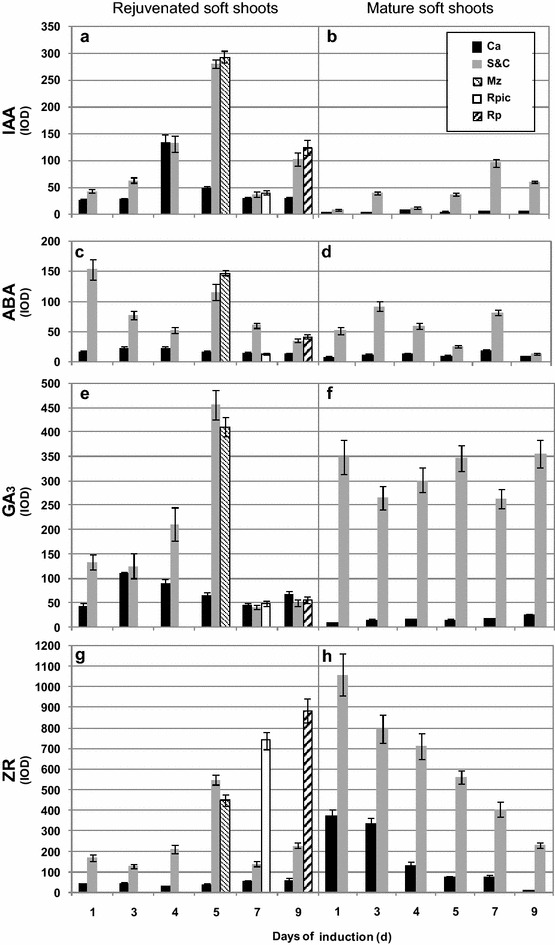
Integrated optical density (IOD) changes in rejuvenated and mature soft shoots. Rejuvenated soft shoots (**a**, **c**, **e**, **g**) and mature soft shoots (**b**, **d**, **f**, **h**) reflect changes in IAA, ABA, GA_3_ and ZR levels during ARF. *Ca* cambium, *S*&*Cs* sieve and companion cells, *Mz* meristem zone, *Rpic* root primordia initial cells, *Rp* root primordia

Figure 7 lists semi-quantitative measurements of the levels of the four endohormones in rejuvenated and mature soft shoots based on the immunolocalization data, and shows the changes in these levels during root induction. The levels of all four endohormones in the S&Cs of rejuvenated and mature soft shoots were significantly different. In the S&Cs of rejuvenated soft shoots, the IODs of IAA and ABA were 43.29 and 153.51, respectively, thus being higher than those of mature soft shoots (IAA 9.55, ABA 52.95). However, in the S&Cs of rejuvenated soft shoots, the IODs of GA_3_ (132.63) and ZR (170.34) were significantly lower than those of mature soft shoots (GA_3_ 348.50, ZR 1059.88).

During rooting induction, IAA, ABA, and GA_3_ levels in the cambium and S&Cs of mature soft shoots did not change significantly, whereas the ZR level gradually decreased. However, the levels of all four endohormones in the cambium, S&Cs, adventitious root meristems, and initial root primordial cells of rejuvenated soft shoots significantly changed over time. In the cambium cells, the changes in IAA and GA_3_ exhibited similar trends, peaking rapidly at days 1–4. The IOD of IAA increased from 26.07 to 134.03, and that of GA_3_ increased from 44.00 to 110.57 but then decreased to 88.87. However, ABA and ZR levels essentially did not change. In S&Cs, the trends of changes in IAA, GA_3_ and ZR levels were consistent, exhibiting gradual increases until peaking on day 5, and then decreasing. The ABA level first fell, then increased, and finally decreased again. On day 1, the IOD of ABA was 153.51, then it fell to 52.63 by day 4, increased to 115.62 by day 5, and then decreased again. As the adventitious root meristems formed, the levels of all four endohormones significantly increased in this tissue, the IODs of IAA, ABA, GA_3_ and ZR became 283.88, 147.96, 410.38 and 451.13, respectively. As the adventitious root meristems developed into adventitious root primordia, the levels of IAA, ABA and GA_3_ gradually decreased, whereas that of ZR gradually increased.

## Discussion

### Rejuvenation and soft-shoot rooting

Juvenile trees usually have vigorous, upright growth habits and exhibit strong resistance to stress, and their rooting ability is powerful. As trees mature, these characteristics are gradually lost, and the ARF capacity of cuttings gradually decreases [41]. Certain rejuvenation treatments, such as repeated subculture in vitro, grafting onto juvenile rootstock, application of growth regulators, heavy pruning, and cutting to promote coppice shooting or root tiller development, may restore or improve ARF of mature trees. For example, Giovannelli and Giannini [19] found that the rooting rate increased from less than 10–70% in mature *Castanea sativa* grafted four times onto juvenile rootstocks, followed by 24 explant subcultures in vitro. Falasca et al. [15] reported that the walnut rooting rate was only 12% after 4 years of subculture in the presence of IBA. However, placing samples in the dark prior to rooting induction can significantly improve the rooting rate [48]. Petridou and Voyiatzis [40] reported that the rooting rate of the difficult-to-root cultivar Kalamon of *Olea europaea* reached 86% when harvested softwood cuttings were subjected to mound-layering. Upon such treatment, the rooting rate of 3-year-old walnut seedlings can attain 40% [49]. In the present study, we showed that a combination of grafting and burying of stock plants horizontally effectively improve ARF of mature walnut plants, the rooting rate of soft shoot cuttings reached 98.2% (Fig. 1e). This method has also been successfully used to rejuvenate *Castanea sativa*, *Diospyros kaki* and *Liriodendron chinensis*, not only *Juglans regia*, with rooting rates greater than 90% in the first three species. The method thus commonly improves ARF of mature trees.

### Organizational structure changed by rejuvenation

We also found rejuvenation induces changes in the organizational structure of mature soft shoots. The most obvious change is that the extent of the sclerenchyma diminishes, and the tissue becomes discontinuously distributed (Fig. 2b, g). Some previous studies have suggested that the formation of discontinuous sclerenchyma tissue is beneficial to rooting, the treatment of etiolation will lead to discontinuous sclerenchyma tissue [33], and the cultivar differences also result in similar results [1]. Our findings indicate that there may be a link between the continuity of sclerenchyma tissue and the plant age (i.e., rejuvenation and mature).

### Soft-shoot rooting and endohormone characteristics

The levels of endogenous hormones in soft shoots changed after grafting and horizontal burying of stock plants. Our immunolocalization data showed that IAA, ABA, GA_3_ and ZR were mainly distributed in phloem S&Cs of soft shoots (Figs. 3a, 4a, 5a, 6a). Earlier studies have shown that IAA is mainly distributed in cambium cells during the rooting induction of the poplar petiole [12, 13] and in the cambium cells of easy-to-root *Eucalyptus grandis* [9]. The distributions of IAA and other hormones in the tissues of soft shoots may reflect the ability of trees to form adventitious roots. During rooting induction, IAA that accumulates in the cambium cells of poplar or eucalyptus acts directly on these cells. In the soft shoots of walnut, the endohormones are mainly distributed in phloem S&Cs and thus cannot act directly in cambium cells, which may explain why walnut is more difficult-to-root than poplar or eucalyptus.

The levels of IAA and ABA were significantly higher in rejuvenated soft shoots S&Cs of walnut than in mature soft shoots, whereas the opposite was true for GA_3_ (S&Cs) and ZR (S&Cs and cambium) in soft shoots (Fig. 7). Endogenous hormonal status and associated molecular mechanisms are altered when mature cuttings are grafted onto juvenile rootstock, for example, after grafting, the IAA content of new shoots germinating from walnut scions increases [44]. In addition, changes in branch polarity affect the distributions of endogenous hormones, and consequently, lateral bud germination and the hormone levels of new shoots. The original polar gradients of endogenous IAA and GA were changed after branch bending in apple [56], walnut [52], and plum trees [6]. Hormonal spread from the shoots to the base aids the germination of lateral and hidden buds, and the IAA and GA contents decrease in the shoot apices, whereas the ABA and ZR distributions exhibit opposite patterns [56]. Mound-layering affects the physiological state of the hazelnut after bud germination. We used in situ hormonal evaluations to show that the hormone levels of rejuvenated soft shoots of walnut changed upon grafting and horizontal burying of stock plants. The level of endogenous IAA, which aids rooting, rose, whereas the level of GA_3_, which reduces rooting, decreased. Thus, rejuvenation affected ARF by varying the hormonal levels in soft shoots.

### The spatial and temporal modifications of endohormones by rejuvenation to improve rhizogenesis

The levels of hormones, particularly IAA, outside phloem S&Cs, especially in the outer layer of the cambium, increased significantly upon rejuvenation (Figs. 3c, d, 7a). IAA accumulation in the cambium is the principal trigger of improvements in the rooting ability of soft shoots of walnut. During rooting induction, the four hormones of mature soft shoots were concentrated in the S&Cs, being rare in other tissues. In rejuvenated soft shoots created by grafting and horizontal burying of stock plants, GA_3_, IAA, ABA and ZR soon appeared in tissues other than phloem S&Cs. By day 4 of rooting induction, the endohormones were present in the outer layer of cambium parenchymal cells opposite the pith (Figs. 3c, 4b, 5c, 6b). However, it remains unclear whether the increased levels of endohormones outside phloem S&Cs reflect hormonal transport from other tissues or synthesis in situ. Some authors have suggested that ARF is closely associated with polar hormonal transport. Dong et al. [12, 13] found that ARF is significantly inhibited by an auxin transport inhibitor (TIBA, 2, 3, 5-triiodobenzoic acid), and a later study showed that TIBA affected the IAA accumulation in the vascular bundles and explant base. Thus, it is possible that the high IAA concentration required for ARF is attributable to transport rather than in situ synthesis. Sukumar et al. [45] found that ARF in *Arabidopsis thaliana* hypocotyls requires intact shoot apical meristems and polar IAA transport. Dawood et al. [7] showed that an auxin polarity transport inhibitor (NPA, N-1-naphtylphtalamic acid) significantly reduces the number of adventitious roots produced by *Solanum dulcamara* and prolongs the duration of ARF.

Based on our results, hormonal transport may have a role. During rooting induction of rejuvenated soft shoots in walnut, GA_3_ appears earlier in tissues outside of phloem S&Cs than do IAA and the other endohormones (Figs. 3b, c, 5b, c). This may affect the transport and distribution of the other endohormones, particularly IAA. GAs may improve stem IAA levels by stimulating polar auxin transport [3]. In a study on GA_3_/IAA interactions, Willige et al. [54] found that GA_3_ affected the level and distribution of the PIN protein on the cell membranes of root meristems in *Arabidopsis thaliana*, changing the IAA distributional pattern and affecting root gravitropism. Similarly, in a study on *Arabidopsis thaliana* root gravitropism, Löfke et al. [30] found that GA_3_ played an important role in controlling auxin location, polar transport and the carrier PIN protein level in the plasma membrane. During root gravitropism, the auxin accumulation in developing roots required the asymmetric distribution of GA_3_. In tobacco (*Nicotiana tabacum*) plants expressing GA_3_ either constitutively or in specific tissues, the precise localization of GA_3_ in the stem is required to regulate the adventitious root development. The emergence of root primordia requires a certain level of GA_3_, the signaling of which is removable [35]. Physiological studies have shown that interactions between GAs and IAA play important regulatory roles in plant development. During adventitious root induction, the fact that GAs accumulate in the cambium earlier than other endohormones may be of a biological importance, and deserves a detailed study.

IAA plays a principal role in adventitious rooting [37, 43]. In this study, IAA became concentrated in the cambium of rejuvenated soft shoots that had thickened 4 days after cutting, mainly in the outer parenchymal layer opposite the pith rays (Fig. 3c). As the root meristems developed, IAA became concentrated in the root primordial cells 5 days after cutting (Fig. 3d). De Almeida et al. [9] have suggested that IAA transforms cambium cells into root primordia. Liu et al. [29] and Hu and Xu [20] have proposed that two main cellular transition steps are required to establish root primordia during de novo root organogenesis in Arabidopsis leaf explants. During root primordia formation, WOX11/12 and WOX5/7 are key genes, and auxin acts upstream of *WOX5/7* and the polar auxin transport to the wounded region is required for the activation of *WOX5/7* by *WOX11/12*.

ABA, GA_3_ and cytokinins influence ARF by interacting with IAA [55]. ABA is required for ARF, but high ABA levels inhibit this process [26, 31]. Tartoura [46] applied exogenous ABA to *Vigna radiata* cuttings during the initiation of ARF and found that the IAA peak was delayed and the length of rooting induction was extended. In our study, the ABA level was always low in the cambium of rejuvenated soft shoots (Fig. 7c). Huang et al. [24] measured ABA contents (using an enzyme-linked immunosorbent assay) during rooting in *Buxussinica* var. *parvifolia*. Prior to the rooting initiation, the ABA level gradually decreased in continuous successive generations and reached a lower concentration compared to other hormones. Generally, it is believed that high GA levels inhibit ARF [4]. Our finding showing that the GA_3_ level was lower in the cambium of rejuvenated soft shoots than in mature soft shoots during the induction of root primordia is consistent with this view (Fig. 7e, f). In a hybrid aspen, Mauriat et al. [32] found that plants overexpressing the key gibberellin biosynthesis gene *AtGA20ox1* grew rapidly but exhibited poor rooting efficiency. GAs appears to compromise ARF by modulating the polar auxin transport. Cytokinins are thought to inhibit rooting by interacting with auxin. In our study, the ZR level was low in soft shoots after rejuvenation but clearly higher during the period of formation of cells of the root primordia, no change was evident prior to this period (Fig. 7g). In a study on Arabidopsis ARF, Della-Rovere et al. [11] found that cytokinins affected auxin biosynthesis and transport by inhibiting the *LAX3* and *PIN1* expression, thus regulating the formation of the quiescent center of the root tip, consistent with the presence of ZR in the central cells of the root primordia in the soft shoots of walnut.

### Endohormone model of rejuvenation in Juglans tree rhizogenesis

**Fig. 8:**
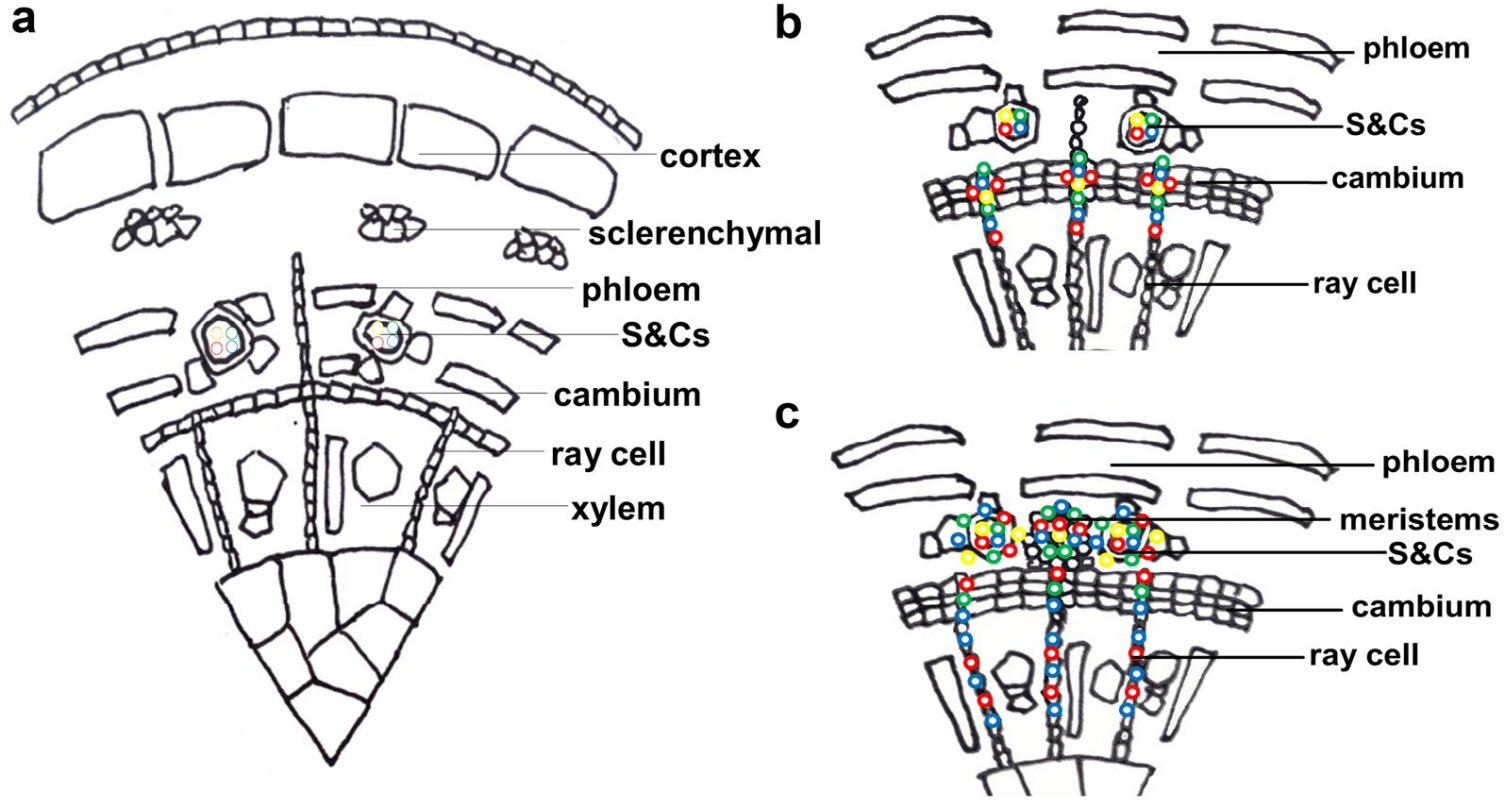
A model of phytohormone distributions during the formation of root primordia. The critical periods of adventitious root induction were 1 day (**a**), 4 days (**b**), and 5 days (**c**). **a** At 1 day of induction, all four types of phytohormones were mainly distributed in S&Cs. **b** As the cambium thickened, the phytohormones were transferred to the cambium cells. GA_3_ and ZR were also present in ray cells. **c** When the meristem cells began to rapidly divide, the four types of phytohormones were mainly distributed in meristems. In addition, the GA_3_ and ZR levels rose further in ray cells. *S*&*Cs* sieve and companion cells, green color = IAA, yellow color = ABA, red color = GA_3_, blue color = ZR

The in situ analysis allowed us to propose how endohormones improve the rooting ability of rejuvenated soft shoots of walnut (Fig. 8). Rejuvenation enhances the IAA level in the S&Cs of soft shoots, and notably elevates endohormone accumulation by the outer cambium cells during rooting. Specifically, during early root induction, the four endohormones are essentially confined to phloem S&Cs. The levels of GA_3_, IAA and ZR increase in the outer cells of the cambium and the cambium becomes thicker. Next, the four endohormones become concentrated in the outer parenchyma cells of the cambium, opposite the pith rays, and these cells divide and differentiate to form root meristems. Finally, the endohormones are present in this tissue and the root meristems develop into root primordia. However, technical constraints prevented us from defining the temporal and spatial features of endohormones distributions in root meristems. This is essential knowledge when trying to understand how endohormones regulate ARF.

## Methods

### Plant material and root induction

We used the walnut cultivar ‘Zhongningsheng’ [ZNS (*Juglans hindsii* × *Juglans regia*)]. The stock tree was 23 years old, had exhibited steady reproductive growth and is located in the Luoning county, Henan province. Rejuvenated soft shoots of ZNS were obtained as follows: first, new ZNS shoots from the stock tree (scions) were grafted to rootstock (1-year-old seedlings). Then, 1-year-old grafted plants were brought into the greenhouse and placed horizontally under 4–5 cm of clean wet sand to ensure maximum shoot bud break. The humidity was held at 50%, the greenhouse used natural light, and the sand temperature was held at 15 ± 5 °C. After 60–70 days of culturing, the rejuvenated soft shoots were harvested. Mature soft shoots similar to the rejuvenated shoots were collected from the 23-year-old stock tree.

Both of the soft shoots bases were dipped in the rooting induction solution [9 mM IBA in 30% (v/v) ethanol in water] for 30 s and submerged 6–8 cm in the root development medium. The shoots were exposed to natural sunlight. The humidity was maintained at about 80% by automatic intermittent spraying. The temperature of the root medium was maintained at 23 ± 5 °C to allow rooting induction. The rooting rates were measured 5, 10, 15, and 20 days after rooting induction. A minimum of 30 soft shoots were collected at each time point to measure rooting rates. All results are expressed as means (± standard errors) of data from at least three replicate experiments. Adventitious roots were directly counted in 5–40 mm regions of basal shoots.

### Morphological and anatomical observations

Ten basal segments (each 1 cm in length) of soft shoots were harvested every day for 15 days after rooting induction. The shoots were immediately washed with distilled water, wiped and divided into two groups. All materials were immediately fixed in FAA [freshly prepared 5% (v/v) formaldehyde, 5% (v/v) acetic acid, and 70% (v/v) ethanol] for at least 24 h, dehydrated through a series of ethanol baths [70, 85, 95, and 100% (all v/v)], cleared in dimethylbenzene and embedded in paraffin. Transverse Sects. (8 μm in thickness) were prepared using a rotary microtome (RM 2245RT, Leica, Wetzlar, Germany), transferred to glass slides, dried overnight, stained with 0.1% (w/v) toluidine blue (Sigma, St. Louis, MO, USA) for 1 min, rinsed in distilled water, dried, dewaxed in xylene and mounted. Photomicrographs were taken (BX41-12P02 instrument, Olympus, Tokyo, Japan). Three replicates of each sample were evaluated.

### In-situ endohormone assays

All endohormones immunolocalization in serial sections were performed using the same microscopic phase. Antibodies were used to localize endogenous IAA, ABA, GA_3_ and ZR during rhizogenesis, using an immunohistochemical approach. Excised samples (approximately 5 mm in length) were rapidly immersed in a freshly prepared 2% (w/v) aqueous solution of 1-ethyl-3-(3-dimethylaminopropyl) carbodiimide (EDC, Sigma Chemical, St. Louis, MO, USA) for 2 h under vacuum (-0.1 Mpa), post-fixed overnight in a solution of 4% (v/v) paraformaldehyde and 2% (w/v) glutaraldehyde at 4 °C, rinsed in phosphate buffer (0.2 M, pH 7.2), and dehydrated in a graded series of ethanol baths [70, 85, 95, and 100% twice (v/v)]. For immunohistochemical observations under a light microscope, all samples were embedded in paraffin and 8 μm thick transverse sections were prepared using the rotary RM 2245RT microtome. The sections were transferred to glass slides, dried overnight at 37 °C, deparaffinized with xylene, and hydrated in a series of ethanol/water baths. The immunolocalization of endohormones was achieved using the method of Gao et al. [17], with slight modifications. The slides were incubated for 15 min in a blocking solution [0.05 M Tris–HCL pH 7.6, 0.3% (v/v) Triton X-100 (Sigma-Aldrich), 10% (v/v) normal goat serum and 5% (w/v) bovine serum albumin], and then kept for 2 h at 37 °C with primary antibodies against IAA, ABA, GA_3_, or ZR [polyclonal bovine serum albumin (Sigma-Aldrich) conjugates, Agrisera AB, Vännäs, Sweden]. Subsequently, the sections were briefly washed [0.05 M Tris–HCL buffer pH 7.6 (TBS), 0.3% (v/v) Triton X-100], blocked once again and incubated for 1 h at 37 °C with gold-labeled goat anti-rabbit IgG or goat anti-rat IgG (Sigma-Aldrich) diluted 1:50 in the TBS/BSA solution. After washing, all sections underwent the silver-enhancement reaction in an appropriate staining solution [0.1 M citrate buffer (pH 3.5), 1.7% (w/v) hydroquinone, 0.1% (w/v) silver nitrate, 5% (w/v) acacia]. As the color developed, the sections were rinsed twice in water, dehydrated, mounted, observed, and photographed using a microscope (BX51-DP25, Olympus).

The negative controls were as follows: first, EDC was omitted during prefixation, second, the primary antibodies were omitted, and third, the secondary antibodies were omitted (see Additional file 1: Fig. s1). All other procedures were identical to those described above. Three replicates of all samples were evaluated. Under the light microscope, any red dots were eliminated as false-positive results.

### Semi-quantification of endohormone levels

The silver particles revealed the distributions of endohormones in soft shoots. To quantify these distributions, the densities of silver particles were measured by observing 30 visual fields of each tissue under an oil lens (×100 objective lens, ×10 ocular lens, BX51-DP25 & DP2-BSW lenses, Olympus). In situ immunolocalization signals were detected using automated image processing software. The mean integrated optical densities (IODs) of silver particles were quantified using Image-Pro Plus (version 6.0, Media Cybernetics Inc. Bethesda, MD, USA). The IODs of silver particles were measured, as described by Taylor and Levenson [47] and Wang et al. [51], with slight modifications. Three repeated measurements were performed for all slides. All data were subjected to the analysis of variance. A P value < 0.05 was considered to reflect a significant difference.

## Additional file

**Additional file 1.** This file contains two figures as follows. **Figure S1.** Controls of endohormone immunolocalization technique. **Figure S2.** Comparison of Fig. 6a and f.

## Abbreviations

ABA: abscisic acid
ARF: adventitious root formation
CTK: cytokinin
EDC: 1-ethyl-3-(3-dimethylaminopropyl) carbodiimide
FAA: formalin–acetic acid–alcohol
GA: gibberellin
IAA: indole-3-acetic acid
IBA: indole-3-butanoic acid
IOD: integrated optical densities
S&Cs: sieve and companion cells
ZR: zeatin riboside
