# Supplementary material for: A novel rejuvenation approach to induce endohormones and improve rhizogenesis in mature Juglans tree
Source: Plant Methods. 2018 Feb 9;14:13. doi: 10.1186/s13007-018-0280-0 (PMC5806478; doi:10.1186/s13007-018-0280-0)
Supplement: Supplementary file 1 — Additional file 1. This file contains two figures as follows. Figure S1. Controls of endohormone immunolocalization technique. Figure S2. Comparison of Fig. 6a and f. [file 13007_2018_280_MOESM1_ESM.docx]

**Supporting information**

Controls for immunolocalization of endohormones

IAA, ABA, GA_3_ and ZR were present in stem tissues during adventitious root induction. We verified the accuracy and specificity of the immune colloidal gold technique by applying several control procedures to sections from the basal regions of soft shoots (rooting induction 1d and 4d) and root primordia. Hormonal signals were minimal when EDC pre-fixation was omitted (Fig. S 1a), indicating that EDC is essential. In addition, no signal was observed when primary (Fig. S 1b) or secondary (Fig. S 1c) antibodies were omitted. Thus, the silver grains reflected antigen-antibody interactions in tissue sections.

**Fig. S 1** Controls of endohormone immunolocalization technique.

Transverse section of the walnut (*Juglans*) tree shoot stained without 1-ethyl-3-(3-dimethylaminopropyl)-carbodiimide (EDC) pre-fixation.

Transverse section of the walnut adventitious root stained without the primary antibody.

Transverse section of the root primordium stained using normal mouse serum instead of primary antibody. Only little IAA, ABA, GA_3_ and ZR signal was detected in any of the control sections. ca = cambium, co = cortex, ph = phloem, pi = pith, rp = root primordia, xy = xylem. Bars: 200 μm.

Comparison of immunolocalization of ZR between rejuvenated soft shoots and mature soft shoots.

The statistics of Figure 7 is based on the same pictures of magnification (100x). The magnification of (a) and (f) in figure 6 is different, so it seems that the signal in (a) obvious. However, under the same magnification there is a difference of the hormone signals in the figure 6a and 6f (Fig. S 2 a). Comparison of figures can be seen mature soft shoots have more ZR signals (Fig. S 2 b).

**Fig. S 2** Comparison of figures 6 a and 6 f.

Figure 6 a is a magnified image of the red box in left figure. Figure 6 f in the revised manuscript (right figure).
